# Racial Inequalities in the Health Establishment Access to the Treatment of COVID-19 in Brazil in 2020

**DOI:** 10.1007/s40615-023-01866-1

**Published:** 2024-01-08

**Authors:** Lídia Santos Silva, Raphael Barreto da Conceição Barbosa, João Paulo Lima, Julio Castro-Alves, Marcelo Ribeiro-Alves

**Affiliations:** 1 National Institute of Infectology Evandro Chagas (INI), Oswaldo Cruz Foundation (FIOCRUZ), Rio de Janeiro, RJ Brazil; 2 Laboratory of Clinical Research On STD/AIDS, National Institute of Infectology Evandro Chagas (INI), Oswaldo Cruz Foundation (FIOCRUZ), Rio de Janeiro, RJ Brazil

**Keywords:** COVID-19, Race factors, Health services accessibility, Hospital units, Systemic racism, Brazil

## Abstract

The Brazilian health system simultaneously allows for the existence of the public and private sectors, which often imposes financial barriers to access to services and affects the health of exposed groups. Studies have shown evidence of higher lethality risks among Black/Biracial and Indigenous People admitted to hospitals due to COVID-19 during the pandemic when compared to White People. This paper evaluated the association between access to treatment for COVID-19, race, and COVID-19-related deaths among the five macro-regions of Brazil in 2020. We conducted a retrospective, cross-sectional observational, and population-wide study. Logistical models were used including first-order interactions between race and the health establishment administration sector using deaths as outcome, adjusted for covariates. The lethality risk, defined as the percentage of deaths among hospitalized patients, of Black/Biracial and Indigenous People was up to 78% (in the Midwest) and 29% (in the South) higher when compared to White People, respectively. The association of the race/access interaction with COVID-19-related deaths suggested the possibility of institutional racism in health establishments. The results highlight the need to guarantee adequate funding to the public health sector to improve equity in access to healthcare and the constant development of educational activities and increased participation of racialized minorities in the healthcare workforce at influential positions for health workers on topics such as racism.

**Supplementary Information:**

The online version contains supplementary material available at 10.1007/s40615-023-01866-1.

## Introduction

Since the 1988 constitution, the Brazilian State is responsible for providing and financing all health services, aiming at universal and equitable access to health services. To meet the new role of the State, the Unified Health System (“*Sistema Único de Saúde*”, SUS) was created. The SUS provides a range of healthcare services, encompassing primary care, emergency services, medical specialties, hospital treatments, medications, mental health care, women’s, children’s, and elderly health, dentistry, disease prevention and control, transplants, and more. Its core goal is to ensure universal, comprehensive, and cost-free access to medical care, promoting health and preventive measures. According to the principle of equity, access to health services should occur correspondingly to the need for care regardless of the socioeconomic status of individuals [1, 2]. However, this new framework of the Brazilian health system also permitted the existence of private health insurance coverage through the supplementary health sector, which allowed double entry into the system mainly for more privileged social segments. With the possibility of access to the public and private healthcare sectors, this institutional design has been fostering health inequality among macro-regions and social segments throughout the country [3]. Although these inequalities are manifested by several dimensions of socioeconomic status [4], the racial dimension of access to healthcare remains a relatively understudied issue due to the lack of data on skin color and ethnic origin in health surveillance systems—its absence being the product of an institutional racism practice [5–7].

Racism is a social determinant of the health-disease process resulting from how, in the course of history, the social structure has been organized in an unequal way and with a critical racial marker as an expression of this inequality, reserving the Black and Indigenous Populations an accumulation of social and economic disadvantages. This means that racism is an expression of domination: concentration of power, which guarantees privileges in the hands of a particular racial group and the accumulation of disadvantages in other groups. In health care, racism operates within the institutions/establishments, hindering the access of certain groups and further accentuating inequities in health [8]. Access barriers can present themselves in different ways [9], e.g., (i) *geographical barriers*, related to the unavailability of services in specific locations and the need for displacement. Initially, the preexisting geographical distribution and the disparate access to Brazil’s healthcare systems significantly influenced the COVID-19 burden. Concurrently, the distribution of the Black/Biracial population aligns with the socioeconomic segment distribution. The most affluent macro-regions, South and Southeast, prioritize private health insurance, disproportionately affecting the less affluent macro-regions, North and Northeast. The private health insurance increases the SUS health coverage disparities beyond per capita income among the macro-regions. Private health services seek to offer services to the strata of the population that can pay for them, thus concentrated in the country’s richest regions, whereas in poor regions, the SUS is the most prevalent health service available. The distribution of the ICU (Type 3)—used to treat severe SARS-CoV-2 infections—among macro-regions was uneven, mainly centered on the wealthiest Southwest and South macro-regions. The South and Southeast have a prevalence rate of 2 and 1.5 ICUs per 100,000 inhabitants, respectively, while in the North, this prevalence rate is 0.03 [10]. This imbalance in ICU coverage across the Brazilian territory imposes geographic barriers to accessing ICUs. In addition, despite the number of ICUs being approximately equivalent in public and private health nets, just after the beginning of the pandemic, the private-sector net expanded the availability of ICUs (Type 3) at a rate faster than the public-sector net [11], increasing the ICU availability gap between the two administrative sectors; (ii) *financial barriers*, which separate those who can afford to pay for certain health services, from those who are entirely SUS dependent. Only 28.5% of the Brazilian population has medical or dental private health insurance [12]. Similarly, considering people with access to private health insurance, 41.5% are Black/Biracial, and 38.8% are White [12], whereas in the general population, 56.2% are Black/Biracial and 42.2% White [13]. It is important to emphasize that the term “financial barrier” is polysemic, and in this study, it will be used to represent aspects of the demand side, i.e., how beneficiaries face economic hurdles to access private ICU beds, generally of better quality of care compared to public ones. Furthermore, during the period of our study, we witnessed the collapse of ICU beds in the public healthcare network, while in the private sector, there were still available beds; (iii) *cultural barriers*, as is the case of institutional racism, which manifests itself through discriminatory norms or behaviors adopted within these institutions. For Almeida (2019) [14], the institutional perspective of racism breaks with the idea that this phenomenon manifests itself only from individual behaviors. In this way, racism is understood as a result of the very functioning of institutions, which “begin to act in a dynamic that confers, albeit indirectly, disadvantages and privileges based on race.” This happens because institutions are hegemonized by certain racial groups that institutionalize their interests through discriminatory mechanisms or even by omission in the adoption of remedial actions. In the health sector, we must consider that this hegemony is also expressed in the professional-user relationship, where power is concentrated in the hands of professionals. We will be employing the concept that racism within healthcare services is a phenomenon that can manifest on both individual and institutional levels, ultimately affecting the health outcomes of ethnic and racial minorities. In the international literature addressing the disproportionate impacts of COVID-19 on racial minorities, we can reinforce the assertions contained in our analysis that structural racism is a significant driver of health disparities. However, unlike the situation in the USA, for example, where Black individuals represent 12% of the population and experienced a 2.1% higher mortality rate than White individuals during the pandemic, in Brazil, Black people constitute the majority of the population (56.2%) while being a minority in terms of economic representation, cultural influence, and political representation. This minority status serves as a social determinant in the health-disease process, as we have observed during the COVID-19 pandemic [15–17].

Several descriptive studies have been dedicated to showing how socioeconomic status, such as age, race, gender, income, schooling, and infrastructure of their households and neighborhoods, have been shaping individuals’ exposure and susceptibility to COVID-19, acting against exposed and marginalized groups [18–21]. Sansone et al. (2022) [18], studying only Indigenous People, provided evidence of a peculiar risk profile that demanded specific policies to avoid deaths by COVID-19. Silva and Ribeiro-Alves (2021) [19], using data from Rio de Janeiro, Brazil, showed that the higher the household per capita income and schooling levels and the lower the neighbors’ poverty, the lower the risk of dying of COVID-19. Similarly, Baqui et al. (2020) [20] and Hallal et al. (2020) [21] presented evidence of a significant increase in the risk of dying of COVID-19 between Indigenous and Black/Biracial People compared to Whites recognizing regional discrepancies among the Brazilian territory. Recent articles have introduced more in-depth analysis using multivariate models to investigate the relationship between the socioeconomic status dimensions that increase the risk of death considering racial groups [22–24]. They found that the higher mortality of Indigenous and Black/Biracial People, when compared to Whites, can, in part, but not totally, be explained by distinct demographic profiles, uneven/unfair preexisting health conditions among races (number of comorbidities), clinical severity on arrival at the health establishment, and the location of the health establishment (geographical barriers to access). Even considering the disadvantaged socioeconomic status of Indigenous and Black/Biracial People, there is still a residual risk of death that cannot be explained by any other reason than race, and due to this, it is attributed to structural racism. Other descriptive studies using ecological data explored the relationship between risk of death, race, and access to health services during the COVID-19 pandemic. Nassif Pires et al. (2021) [25] highlighted the possibility that structural inequalities could affect access to healthcare, punctuating the potential interconnection between race, the geographical distribution of health services, and income inequality to aggravate the pandemic. Tavares and Betti (2021) [26] indicated that considering factors previously pandemic, Black/Biracial and Indigenous People were at higher risk of death when compared to Whites, whether due to poverty, health conditions, or access to health services. However, there are still no studies that show the magnitude of the barriers to financial access to health services that, in fact, may have reinforced racial inequalities in the risk of death during the epidemic, as well as analyzing death by racial groups considering the financial barriers to accessing health services.

In this paper, using individual population-based data, we propose a reflection on the relationship between race, financial access to treatment for COVID-19, and lethality, overall and among the five Brazilian macro-regions. Our results suggest the existence of distinct risks of death associated with financial barriers, as well as cultural barriers to access in the public health net and the private-sector net probably manifested by institutional racism.

## Methods

### Data

#### Health Establishments (CNES)

Health establishments data were extracted from the base of the National Registry of Health Establishments (CNES), available at http://cnes.datasus.gov.br/, referring to December 2020; period of registration from the notification of the first case to the last report of cases prior to the start of the vaccination campaign against SARS-CoV-2 to avoid introduce another important confounding, the vaccination, which the access would also be disproportionate among different racial and social groups. We extracted the administration sector (“public” and “private or philanthropic”) to which each health establishment belonged.

#### COVID-19 Microdata (SIVEP)

COVID-19 pandemic data was extracted from the Influenza Epidemiological Surveillance Information System (SIVEP-Gripe) database, available at https://opendatasus.saude.gov.br/dataset/srag-2021-a-2023. SIVEP-Gripe is an official open-source national surveillance database used to monitor all severe acute respiratory infections and resulting hospital admissions in Brazil. SIVEP-Gripe data is census data. It is established and maintained by the Brazilian Ministry of Health (MoH, Brazil) and contains individualized data on notifications of severe acute respiratory syndromes, including those caused by the SARS-CoV-2 infection. More specifically, we extracted from this base the information of patients aged 20 years or older who had their first symptoms by 2020-12-31 with a confirmed diagnosis of COVID-19. We extracted the variables age (“18–40,” “40–60,” “60–80,” “>80”), gender (“female,” “male”), race (“White,” “Black/biracial,” “Indigenous,” “Others”), schooling (“illiterate,” “incomplete fundamental,” “complete fundamental,” “high school,” “college”), comorbidities (number of comorbidities, “0,”“1–2,” “3–4,” “>4”), residence in the same municipality of the health establishment (i.e., whether the patient was treated in the same municipality of residence), report delay (i.e., the number of days between the first symptoms and the notification of the SARS-CoV-2 infection), death, and urban region (“urban,” “rural/unk”).

#### Race

We chose to use the *Instituto Brasileiro de Geografia e Estatística* (IBGE) categories for self-declaration on the race/color question, i.e., black, brown, white, red/indigenous, and yellow, with modifications. The concept of race in a mixed population like Brazil does not exactly match the definition of race in other studies or countries. For instance, the category Black/Biracial as the sum of black and brown people is widespread in Brazil and is defined in the Statute of Racial Equality (Law No. 12288/2010)—however, as stated earlier, the IBGE (2019b) [27] classifies as brown the descendants of the interracial relationships, a classification that is not very widespread and that differs from the classification present in the Racial Equality Statute, used for affirmative policies such as quotas in universities. Even government agencies such as the Institute of Applied Economic Research (IPEA) use the category “black” as the sum of blacks and browns (IPEA, 2020) [28]. Thus, we opted to recategorize individuals self-declaring as brown to the black category, which we called “Black/Biracial,” and group people who self-declared as yellow and those that ignored the question of race/color as “others.” The use of “Biracial” instead of “brown” is to avoid misunderstandings, for readers unfamiliar with Brazil’s ethnic makeup, between the term “brown” to describe skin color and that of descendants of Middle Eastern ethnic groups, commonly described in the literature as “brown.”

#### Socioeconomic Information

To evaluate patients’ socioeconomic environment, we made use of the variables *Per Capita* Household Income (average monthly family income *per capita*), Gini Index of household income, and “Bolsa Família” Program (BFP) Beneficiary (% of the population of a municipality benefited by the BFP) at municipal-level obtained from the 2010 census (IBGE/Brazil).

### Data Merge

The merge of the CNES and SIVEP datasets was accomplished by utilizing the health establishment code as the linkage variable. The CNES dataset comprised 376,221 health establishments, while SIVEP featured 593,799 records of confirmed SARS-CoV-2 infections. Following the integration of SIVEP and CNES databases, the resulting dataset comprised 5707 establishments and 590,102 individuals. The dataset loss of approximately 3000 admission records due to incorrect health establishment code annotation, a discrepancy representing only 0.67% of the sample, would likely not cause systematic bias. Lastly, the 2010 census data was integrated with these datasets through the utilization of the municipality code. The complete flowchart for selecting the study population can be consulted in the Appendix, Suppl. Figure 1.

### Statistical Analysis

To determine the association between deaths, race, and financial barriers to access (administration sector) in the health establishment to the management of COVID-19, we used generalized linear models with a logit link function and a random effect for the federation units. The dataset was built at the individual level, and the outcome was categorized as either death (“yes”/“no”). Adjustment variables were included as an attempt to mitigate the effect of the sociodemographic and clinical profile of patients [19], geographic barrier, and socioeconomic geographic contextual factors [29, 30] on the estimation of race and financial barriers. Due to this, the models included age, gender, schooling, report delay, and comorbidities as sociodemographic and clinical factors recognized as risk factors for death of COVID-19 hospitalized patients; residence in the same municipality of the health establishments as geographic barrier indicator; and Per Capita Household Income, Gini Index, and BFP Beneficiary as socioeconomic contextual factors. We developed four distinct models using death as the outcome while varying the inclusion of covariates, such as race and financial barriers to access: (I) univariate models, containing race or administration sector; (II) multivariate models, where the race was adjusted for age, gender, schooling, residence in the same municipality of the health establishments, report delay, comorbidities, macro-region, *Per Capita* Household Income, Gini Index, and BFP Beneficiary; (III) the same model mentioned before, including administration sector as an adjusting variable; (IV) multivariate models including a first-order race and administration sector interaction, adjusted by all covariates of the model (II). The rationale behind these models was to explore the association between the exposure variables, race, and financial barriers to access, and the death outcome. These models encompassed the following scenarios: exposure variables separately (univariate models I), exposure variables separately with ordinary controls (multivariate models II), exposure variables together and ordinary controls (multivariate models III), and a first-order interaction between the exposure variables along with ordinary controls (multivariate models IV). Measures of association were presented as odds ratios (OR), odds ratios adjusted for ordinary controls (aOR), and their 95% confidence intervals. All statistical analyses were performed in R v.4.0.5 software, the “lme4” library, and its dependencies.

## Results

The description of the population characteristics of hospitalized patients with COVID-19 is provided in Table 1, focusing on key dimensions of our analysis, including, deaths, race, and the administration sector of the health establishment. The searching of hospitals by patients with COVID-19 in Brazil was very similar between the public (49%) and private (51%) administrative sectors. Nevertheless, fatalities associated with COVID-19 were more prevalent in the public sector (38%) as opposed to the private sector (27%). The racial distribution within our patient population was as follows: White (38%), Black/Biracial (38%), and Indigenous (*n*=1649, < 1%). However, when examining the clientele of private health establishments, Black/Biracial individuals comprise only 26%, whereas Whites constitute 49%—nearly double the percentage. The table also indicates important macro-region differences in the administration sector and racial distribution of the hospitalized patient population. Access to COVID-19 treatment in private healthcare facilities was predominant in only two macro-regions: the Southeast (57%) and the South (75%), which are the most prosperous regions of the country. Concomitantly, these were the macro-regions with more patient declared as White.

**Table 1:** Descriptive analysis of demographic characteristics, clinical severity on arrival at the health establishment, and distance between home and the health establishment by administrative sector, death outcome, and race. Brazil, 2020

		Administration sector		Death		Race			
		Private	Public	No	Yes	White	Black/Biracial	Others	Indigenous
Brazil		301,147 (51%)	288,955 (49%)	399,900 (68%)	190,202 (32%)	225,606 (38%)	221,924 (38%)	140,923 (24%)	1649 (< 1%)
Age	18–40	46,116 (53%)	41,529 (47%)	78,882 (90%)	8763 (10%)	29,960 (34%)	35,111 (40%)	22,260 (25%)	314 (< 1%)
	40–60	102,357 (52%)	92,622 (48%)	155,599 (80%)	39,380 (20%)	71,924 (37%)	74,112 (38%)	48,423 (25%)	520 (< 1%)
	60–80	111,871 (49%)	116,825 (51%)	133,637 (58%)	95,059 (42%)	90,182 (39%)	85,701 (37%)	52,265 (23%)	548 (< 1%)
	> 80	40,803 (52%)	37,979 (48%)	31,782 (40%)	47,000 (60%)	33,540 (43%)	27,000 (34%)	17,975 (23%)	267 (< 1%)
Gender	Female	132,299 (51%)	128,455 (49%)	179,879 (69%)	80,875 (31%)	101,393 (39%)	96,656 (37%)	62,015 (24%)	690 (< 1%)
	Male	168,817 (51%)	160,436 (49%)	219,950 (67%)	109,303 (33%)	124,191 (38%)	125,235 (38%)	78,868 (24%)	959 (< 1%)
	Indeterminated	31 (33%)	64 (67%)	71 (75%)	24 (25%)	22 (23%)	33 (35%)	40 (42%)	0 (0%)
Race	White	146,874 (65%)	78,732 (35%)	154,723 (69%)	70,883 (31%)	225,606 (100%)	0 (0%)	0 (0%)	0 (0%)
	Black/Biracial	76,984 (35%)	144,940 (65%)	142,780 (64%)	79,144 (36%)	0 (0%)	221,924 (100%)	0 (0%)	0 (0%)
	Indigenous	477 (29%)	1172 (71%)	1008 (61%)	641 (39%)	0 (0%)	0 (0%)	0 (0%)	1649 (100%)
	Others	76,812 (55%)	64,111 (45%)	101,389 (72%)	39,534 (28%)	0 (0%)	0 (0%)	140,923 (100%)	0 (0%)
Schooling	Illiterate	4818 (32%)	10,176 (68%)	6771 (45%)	8223 (55%)	5120 (34%)	8871 (59%)	748 (5%)	255 (2%)
	Incompl. Fund	25,957 (46%)	30,875 (54%)	32,049 (56%)	24,,783 (44%)	28,793 (51%)	25,403 (45%)	2349 (4%)	287 (1%)
	Compl. Fund	19,201 (49%)	19,940 (51%)	24,908 (64%)	14,233 (36%)	19,842 (51%)	17,531 (45%)	1655 (4%)	113 (< 1%)
	High School	38,594 (57%)	28,970 (43%)	50,629 (75%)	16,935 (25%)	34,586 (51%)	27,892 (41%)	4971 (7%)	115 (< 1%)
	College	25,972 (75%)	8587 (25%)	27,808 (80%)	6751 (20%)	21,517 (62%)	9406 (27%)	3585 (10%)	51 (< 1%)
	Unknown	186,605 (49%)	190,407 (51%)	257,735 (68%)	119,277 (32%)	115,748 (31%)	132,821 (35%)	127,615 (34%)	828 (< 1%)
Death	No	220,542 (55%)	179,358 (45%)	399,900 (100%)	0 (0%)	154,723 (39%)	142,780 (36%)	101,389 (25%)	1008 (< 1%)
	Yes	80,605 (42%)	109,597 (58%)	0 (0%)	190,202 (100%)	70,883 (37%)	79,144 (42%)	39,534 (21%)	641 (< 1%)
Residence in the same municipality	Yes	210,738 (49%)	216,114 (51%)	291,550 (68%)	135,302 (32%)	166,261 (39%)	162,337 (38%)	97,131 (23%)	1123 (< 1%)
Health establishment	No	90,409 (55%)	72,841 (45%)	108,350 (66%)	54,900 (34%)	59,345 (36%)	59,587 (37%)	43,792 (27%)	526 (< 1%)
Report delay (in days)		9.57	10.93	10.12	10.47	9.09	10.63	11.44	10.35
Number of	0–1	104,534 (49%)	108,235 (51%)	166,639 (78%)	46,130 (22%)	73,197 (34%)	81,992 (39%)	56,760 (27%)	820 (< 1%)
Comorbidities	1–2	136,983 (51%)	129,991 (49%)	173,796 (65%)	93,178 (35%)	105,376 (39%)	99,786 (37%)	61,166 (23%)	646 (< 1%)
	2–4	53,563 (54%)	46,120 (46%)	54,838 (55%)	44,845 (45%)	41,974 (42%)	36,563 (37%)	20,985 (21%)	161 (< 1%)
	> 4	6067 (57%)	4609 (43%)	4627 (43%)	6049 (57%)	5059 (47%)	3583 (34%)	2012 (19%)	22 (< 1%)
Urban region	rural/unk	43,731 (47%)	48,467 (53%)	60,898 (66%)	31,300 (34%)	27,958 (30%)	33,474 (36%)	29,785 (32%)	981 (1%)
	urban	257,416 (52%)	240,488 (48%)	339,002 (68%)	158,902 (32%)	197,648 (40%)	188,450 (38%)	111,138 (22%)	668 (< 1%)
Macro region	Midwest	26,587 (42%)	36,768 (58%)	47,659 (75%)	15,696 (25%)	13,662 (22%)	28,287 (45%)	20,986 (33%)	420 (1%)
	Northeast	40,005 (35%)	73,284 (65%)	68,603 (61%)	44,686 (39%)	12,633 (11%)	64,630 (57%)	35,845 (32%)	181 (< 1%)
	North	9806 (22%)	34,736 (78%)	27,128 (61%)	17,414 (39%)	4335 (10%)	33,711 (76%)	5809 (13%)	687 (2%)
	Southeast	164,036 (57%)	123,424 (43%)	197,815 (69%)	89,645 (31%)	130,095 (45%)	87,756 (31%)	69,407 (24%)	202 (< 1%)
	South	60,713 (75%)	20,729 (25%)	58,686 (72%)	22,756 (28%)	64,875 (80%)	7535 (9%)	8873 (11%)	159 (< 1%)
Administration sector	Private	301,147 (100%)	0 (0%)	220,542 (73%)	80,605 (27%)	146,874 (49%)	76,984 (26%)	76,812 (26%)	477 (< 1%)
Health establishment	Public	0 (0%)	288,955 (100%)	179,358 (62%)	109,597 (38%)	78,732 (27%)	144,940 (50%)	64,111 (22%)	1172 (< 1%)

In summary (Table 1), the overall distribution of COVID-19 cases treated in public and private hospitals was similar. Public establishments, on average, exhibited a higher fatality rate than private ones. However, the patient demographics in these two types of health establishments were quite distinct: the public sector primarily served Black/Biracial and Indigenous populations from the country’s poorest regions, while the private healthcare sector catered to a predominantly White population residing in more affluent regions of the country. Were these factors significant contributors to the higher fatality rate in COVID-19 hospital treatment among Black/Biracial and Indigenous individuals when compared to White individuals? A more detailed examination of this is presented in Table 2 below.

**Table 2:** Associations (OR [95%CI] and aOR [95%CI]) of COVID-19-related death estimated by multiple logistic models in Brazilian macro-regions among levels of race and health establishments administrative sector. Brazil, 2020

		Brazil			Midwest			Northeast		
		(I)	(II)	(III)	(I)	(II)	(III)	(I)	(II)	(III)
Race	White	Reference	Reference	Reference	Reference	Reference	Reference	Reference	Reference	Reference
	Black/Biracial	0.96 [0.95; 0.97]	**1.06 [1.05; 1.08]**	**1 [0.98; 1.01]**	0.97 [0.93; 1.02]	0.99 [0.94; 1.04]	0.97 [0.92; 1.02]	0.93 [0.89; 0.96]	0.97 [0.93; 1.01]	0.93 [0.89; 0.97]
	Indigenous	**1.2 [1.11; 1.3]**	**1.24 [1.11; 1.39]**	**1.2 [1.07; 1.34]**	**2.02 [1.64; 2.47]**	**1.78 [1.41; 2.24]**	**1.78 [1.41; 2.25]**	0.92 [0.73; 1.16]	0.94 [0.68; 1.3]	0.91 [0.66; 1.26]
	Others	0.68 [0.67; 0.7]	0.8 [0.78;0.81]	0.8 [0.78; 0.81]	0.78 [0.74; 0.82]	0.81 [0.76; 0.86]	0.8 [0.75; 0.86]	0.7 [0.67; 0.73]	0.82 [0.78; 0.86]	0.82 [0.78; 0.86]
Administration	Private	Reference		Reference	Reference		Reference	Reference		Reference
Sector	Public	**1.56 [1.54;1.57]**		1.57 [1.55;1.59]	1.24 [1.19;1.29]		1.22 [1.17;1.28]	1.8 [1.75;1.85]		1.74 [1.69;1.79]
		**North**			**Southeast**			**South**		
		(I)	(II)	(III)	(I)	(II)	(III)	(I)	(II)	(III)
Race	White	Reference	Reference	Reference	Reference	Reference	Reference	Reference	Reference	Reference
	Black/Biracial	0.89 [0.84; 0.95]	0.98 [0.91; 1.05]	0.95 [0.88; 1.02]	0.97 [0.95; 0.98]	**1.12 [1.1; 1.14]**	**1.01 [0.99; 1.03]**	**1.25 [1.18; 1.31]**	**1.32 [1.24; 1.39]**	**1.29 [1.22; 1.37]**
	Indigenous	**1.22 [1.04; 1.43]**	**1.41 [1.16; 1.7]**	**1.35 [1.12; 1.64]**	0.57 [0.49; 0.68]	0.62 [0.44; 0.88]	0.62 [0.44; 0.88]	0.99 [0.73; 1.34]	1.27 [0.86; 1.88]	1.26 [0.86; 1.87]
	Others	1.01 [0.93; 1.09]	1.02 [0.93; 1.11]	1.04 [0.95; 1.13]	0.59 [0.57; 0.6]	0.71 [0.69; 0.72]	0.71 [0.7; 0.73]	1.01 [0.96; 1.06]	1.08 [1.02; 1.14]	1.09 [1.03; 1.15]
Administration	Private	Reference		Reference	Reference		Reference	Reference		Reference
Sector	Public	1.29 [1.23; 1.35]		1.47 [1.39; 1.55]	1.62 [1.59; 1.64]		1.67 [1.64; 1.7]	1.32 [1.27; 1.37]		1.18 [1.14; 1.23]

(I) univariate models; (II) multivariate models adjusted for age, gender, schooling, residence in the same municipality of the health establishments, report delay, comorbidities, macro region, Per Capita Household Income, Gini Index, and BFP Beneficiary; (III) model (II), including administration sector as an adjustment variable

Table 2 presents the associations of COVID-19-related deaths in Brazilian macro-regions by race and health establishments administrative sector, considering different ordinary controls. We can observe that in Brazil, there exists a higher crude risk of death for Indigenous People (OR = 1.20 [1.11;1.30], model I), not taking into consideration that Indigenous People live under distinct realities. When we consider these realities (model II), including the socioeconomic (i.e., age, gender, schooling) and clinical (i.e., report delay and comorbidities) profile, as well as the same geographical context (i.e., residence in the same municipality of the health establishments, macro-region, Per Capita Household Income, Gini Index, and BFP Beneficiary), this risk did not disappear (aOR = 1.24 [1.11;1.39], model II). Finally, to assess the relationship of the financial barriers to access private administration sector with changing the risk of death by race, we implemented models (III). Comparing models (II) and (III) in Brazil, access to health establishments (administration sector) was partially responsible for the differential risk of death by race since their inclusion in the model diminished it. In the case of Indigenous People, this risk was decreased (aOR = 1.20 [1.07;1.34]), but it is significant, whereas in the Black/Biracial People, the inclusion of the administration sector equalized the risks in relation to Whites (aOR = 1 [0.98;1.01]).

Nevertheless, given Brazil’s vast geographical dimensions, the cultural diversification and socioeconomic disparities across its regions, we decided to reproduce the national analysis in the different macro-regions. These analyses revealed effects that were not as pronounced when examining the national level. Employing the same logic as in the previous assessment at the national level, Table 2 demonstrates that in the Midwest and North macro-regions, evidence of racial disparities against Indigenous People is apparent, while in the Southeast and South, it is observed against Black/Biracial People.

Table 3 shows the risk of death by the first-order interaction between race and administration sector using as reference the category White People that used the private sector. The first aspect to highlight is something congruent with Tables 1 and 2, i.e., for any race, the risk of death is higher in public establishments when compared to private ones. For example, at Brazil’s level, independently of race, on average, the risk of death was higher in the public sector (Black/Biracial in private versus in public, 1.02 [1;1.05] vs. 1.51 [1.48;1.54]; Indigenous in private versus in public, 1.08 [0.87;1.34] vs. 1.89 [1.66;2.16]; Whites in private versus in public, 1.51 [1.48;1.54]) for people with the same race. The second and most impressive aspect is that, in the public or private sectors, there were differences in the risk of death by race, even considering socioeconomic and clinical profile or the geographical context. At Brazil’s level, in the private sector, the risk of death to Black/Biracial was higher than for Whites (aOR = 1.02 [1;1.05]), as well as in public was higher for the Indigenous People (aOR = 1.89 [1.66;2.16] vs. Whites, 1.51 [1.48;1.54]). Regional scenarios presented even more discrepancies, and again, the Midwest (aOR = 2.69 [2.01;3.61] vs Whites, 1.30 [1.19;1.42]) and North (aOR = 1.88 [1.51;2.34] vs Whites, 1.37 [1.18;1.58]) regions in the public showed a higher risk of death to Indigenous People when compared to Whites, and the Southeast (aOR = 1.03 [1;1.06]) and South (aOR = 1.35 [1.25;1.46]), in the private sector against Black/Biracial People. It is important to stress that this residual risk captured by the race variable, residual in the sense that cannot be explained by any covariate that we used in these models, can be attributed to cultural barriers or the structural racism manifested in the health institutions, public and private ones.

**Table 3:** Associations (aOR [95%CI]) of COVID-19-related death estimated by multiple logistic models in Brazilian macro-regions among first-order interaction of race and health establishments administrative sector. Brazil, 2020

		Brazil	Midwest	Northeast	North	Southeast	South
		(IV)	(IV)	(IV)	(IV)	(IV)	(IV)
Race by	White:Private	Reference	Reference	Reference	Reference	Reference	Reference
Administration	Black/Biracial:Private	**1.02 [1;1. 05]**	0.95 [0.87; 1.03]	0.98 [0.91; 1.05]	0.98 [0.86; 1.12]	**1.03 [1; 1.06]**	**1.35 [1.25; 1.46]**
Sector	Indigenous:Private	1.08 [0.87; 1.34]	1.37 [0.93; 2.01]	1.4 [0.76; 2.55]	1.31 [0.56; 3.08]	0.6 [0.38; 0.97]	0.96 [0.57; 1.63]
	Others:Private	0.73 [0.71; 0.74]	0.91 [0.83; 0.99]	0.82 [0.76; 0.88]	0.78 [0.67; 0.91]	0.63 [0.61; 0.65]	1.06 [0.98; 1.14]
	White:Public	1.51 [1.48; 1.54]	1.3 [1.19; 1.42]	1.82 [1.68; 1.97]	1.37 [1.18; 1.58]	1.57 [1.53; 1.61]	1.15 [1.09; 1.2]
	Black/Biracial:Public	1.51 [1.48; 1.54]	1.26 [1.17; 1.36]	1.64 [1.53; 1.75]	1.3 [1.15; 1.47]	1.61 [1.57; 1.65]	1.24 [1.12; 1.37]
	Indigenous:Public	**1.89 [1.66; 2.16]**	**2.69 [2.01; 3.61]**	1.4 [0.96; 2.06]	**1.88 [1.51; 2.34]**	1.01 [0.62; 1.65]	2.43 [1.15; 5.15]
	Others:Public	1.33 [1.3; 1.36]	0.95 [0.87; 1.03]	1.49 [1.39; 1.59]	1.68 [1.46; 1.94]	1.31 [1.26; 1.35]	1.11 [0.99; 1.23]

^*^Multivariate models adjusted for age, gender, schooling, residence in the same municipality of the health establishments, report delay, comorbidities, macro region, Per Capita Household Income, Gini Index, and BFP Beneficiary

## Discussion

This article questioned whether racial inequalities and financial or cultural barriers in the health establishments influenced the lethality risk of COVID-19 in Brazil. We found a rise of lethality considering both, financial barriers to access—against public establishments—and relevant racial inequalities against Black/Biracial and Indigenous People compared to White. Although observed important regional particularities, the Indigenous People were the most affected, even compared to Black/Biracial individuals. There was strong evidence of higher lethality risks in the public health sector compared to the private one, revealing that the financial barriers to access generated substantial health inequalities during the pandemic. Black/Biracial and Indigenous People are the most frequent users of the public health net and the more exposed to higher risks of death from COVID-19. The distinct access between public and private health sectors was responsible for a fraction of the observed racial inequalities in the lethality risk, and their size varies according to race and region. It was a relevant factor in the South and Southeast to the Black/Biracial People, maybe due to the higher presence of the private sector or the significant presence of White People in the general population. For Indigenous People, the importance of the type of access to health services was more restricted. However, the access showed relevance to explaining the racial differences in lethality risk due to the access to the private and public, not only by the insertion by itself. We also observed that the lethality risk was substantially distinct among races, even considering only patients treated at the same administrative sector health establishments. For Black/Biracial and Indigenous People in the Southeast and South macro-regions, the lethality risk was substantially higher in the private sector compared to White individuals. In the Midwest and North macro-regions, where Indigenous People are more numerous, and most health establishments are public, there was a difference in the lethality risk against this race in the public health net when compared to Whites. It is worth noting that any of the differences described before can be attributed to demographic characteristics, clinical severity on arrival at the health establishment, or distance between home and the health establishment (geographical barriers to access). By exclusion, we are convinced that the results are due to cultural barriers associated with racism. Authors such as Fanon (2018) [31] and Almeida (2019) [14] emphasize racism’s ability to shape subjectivities and highlight this process occurs in two ways. First, through the performance of the cultural industry and the educational system, which historically dehumanize Black and Indigenous populations and present them in a position of incapacity/subordination, and secondly, through the observation of racism expressed in their own material reality in which part of these populations survive in subaltern conditions, on the margins of society. Thus, it is as if reality confirms cultural representations forming a racist social imaginary. As previously mentioned, social conflicts are reproduced inside institutions, since they make up society. In health establishments, it is no different; racism is able to shape the social unconscious, so that “the ‘normal’ life, affections and ‘truths’” are permeated by it, not depending on “a conscious action to exist” [14]. For instance, when considering nursing as a whole, the largest health professional category, 53% of professionals in this class are Black/Biracial. However, if we observe only nurses (higher education professionals), Black/Biracial represent 37.9%, and Indigenous People are less than 10%. Among physicians, Black/Biracial represent less than 20%. Therefore, even with the large representation of Black/Biracial People in the health workforce, institutional racism appears to continue operate. We must consider that higher education professionals generally hold management/leadership positions, mostly White People. Thus, the power that we mentioned at the beginning of this article is concentrated under the domination of a racial group. Therefore, we believe that the main measure to face racism is the reduction of socioeconomic inequalities and the social determination of racism. In the health area, one measure to modify the reproduction of these behaviors is the inclusion of topics such as racism, the health of the Black Population, and the health of the Indigenous Population in the formative processes of health courses and in the continuing education of workers in the area.

Studies using in-hospital data during the pandemic in Brazil showed that Black/Biracial had a higher risk of death and “lower demand for health resources”—or lack of access—when compared to Whites, independently of age, sex, education, and days of symptoms [22]. Similar results were found in Peres (2021) [24] considering the risk of death, which reinforced our findings. Unfortunately, to compare our results beyond the pandemic context, we are faced with a scarcity of open and adequate data in Brazil. There are almost no studies that use analyses similar to those applied here to compare access, race, and health inequalities. However, the existing pieces of evidence already produced are largely in accordance with our results, reinforcing the evidence of cultural barriers and racism in the access to health services. Black women diagnosed with breast cancer who received care both in the private and public sector health establishments in the southeast zone of the sixtieth-largest city in Brazil, Belo Horizonte (MG), had a higher delay from diagnosis to therapy than white or biracial ones, increasing their risk of death [32]. Again, in the second-largest city in Brazil, Rio de Janeiro (RJ), black puerperal women receiving care at a public health establishment had worse prenatal and childbirth care when compared to biracial or white ones [33]; astonishing, even the use of anesthetics during deliveries was lower in these women. In the macro-region highlighted in our results, the South region, more precisely in two large cities, São Leopoldo and Pelotas, in the southernmost state of the country, Rio Grande do Sul, regardless of income and other socioeconomic status variables, the access to early detection exams for breast (mammography) and cervical (cervical cytology pap smear) cancer was lower to black women when compared to white [34]. Regarding the Indigenous People that, since the colonization of Brazil, has its history marked by the violation of its culture and territories, but also by the occurrence of several epidemics that decimated part of its peoples, studies confirm our findings in relation to the greater lethality by COVID-19 among this population when compared to the White Population [35]. The presence of illegal economic activities, especially those that promote deforestation, increases the risk of infection for Indigenous People as they increase contact with miners [36].

The colossal effect on health of financial barriers to access health establishments in Brazil can bring valuable lessons in promoting health policies for beyond the pandemic and deserve further discussion. The pandemic was reproduced during the public health emergency. Exposed segments were strongly affected in terms of the burden of COVID-19, such as deprived macro-regions and historically disadvantaged races. A key point of our evidence was the higher lethality risks in public health establishments compared to private ones. This reality cannot be well evaluated without considering the health financing structure in Brazil. In 2019, the domestic general health expenditure was approximately 9.6% of the Gross Domestic Product (GDP) [37], of which 60% was destined for the private health administrative sector. However, only 28.5% of Brazilians have access to private health insurance [12], evidencing the inequality reality of the access to health services in Brazil. Consequently, the public-sector health net, in addition to being highly underfunded, is also over-demanded, given that, in theory, it is responsible for the care of 71.5% of the Brazilian population [12]. Given this scenario, it is not surprising that health establishments in the public administrative sector had relatively lower service effectiveness when compared to health establishments in the private administrative sector, which was materialized by the difference in lethality risk during the COVID-19 pandemic. Thus, our evidence suggests that an effective measure to shorten the gap between races and regional inequalities in health in Brazil is to increase financing for the public health administrative sector. The pandemic experience emphasized the strong dependence of the public health sector on the most exposed population and in the least assisted macro-regions of Brazil. Unfortunately, we are observing an adverse scenario in the Brazilian health financing structure, especially after the beginning of the implementation of macroeconomic austerity policies adopted in 2015. Although total health expenditures had increased as a proportion of GDP during this period, public financing has reduced its share compared to the private sector. The persistence of this trend can further increase racial inequalities in health.

The study has limitations. Firstly, it is essential to justify our choice to aggregate the results for black and biracial groups. In Brazil, racial discrimination operates based on the phenotype traits of the discriminated group. In this sense, the “mestizo,” for having one or more of the characteristics (skin color, hair, or other traits) of the black, i.e., having some of the phenotypes, is also seen as inferior. After the abolition of slavery in Brazil, white supremacist groups advocating eugenics propagandized that Brazil’s racial problem was not necessarily the blacks, as they represented a small portion of the population, easy to isolate. The problem was the “mestizo,” who they believed to be inferior because they lacked the purity of the races they descended from and also because of their numerical representation and dispute over spaces previously occupied only by whites. Even today, the prejudice intensifies in direct proportion to the color scale and the size of other phenotype traits; the blacker a person is, the more likely they are to be a victim of prejudice [38]. Therefore, the aggregation of blacks and biracial and their designation as blacks is doubly justified. Statistically, because the socio-economic characteristics of the two groups are the same. Theoretically, because the potential or actual discrimination suffered by both groups is of the same nature. In other words, biracial people are discriminated against because they are black. Second, a large share of the COVID-19 suspects did not self-declare as belonging to any race. Twenty percent of our sample comprises the category “other,” which includes “not self-declared” patients. On this topic, it is noteworthy that only in 2017 did the question of race/color become mandatory in SUS forms (Ordinance 344, February 1, 2017). Until then, its use was optional, limiting the production of epidemiological data that would allow analyses that reflect the non-white population’s real health and disease conditions. We can affirm that the nearly three decades (between the creation of the SUS and the publication of Ordinance 344) without collecting race/skin color data were an expression of institutional racism and contributed to maintaining racial inequities in health. Third, we opted to add the data from philanthropic health establishments to those from private health establishments since both have a private sector legal nature, nonprofit and for-profit, respectively. Despite providing a significant volume of SUS services, philanthropic health establishments differ from public ones in several aspects, such as, for example, in the bureaucracy. While public administration health establishments must comply with federal law 8.666/93 on the forms of hiring personnel, companies, and services and on the purchase of inputs and equipment, philanthropic health establishments can perform these transactions with the agility and flexibility typical to any private company. However, it is worth noting that there are ICUs in philanthropic hospitals reserved for SUS, making it impossible to distinguish which type of funding was carried out for each patient. Fourth, racism is not a simple phenomenon to identify and measure; except in the case of using overt racial slurs directed at minority groups, we treated the impact of racism as a residual effect of the “race” variable when alternative explanations for these health outcomes were ruled out, which is, all observed variable was controlled. Fifth, since we are estimating racism as a residual effect of the race/ethnicity variable, its effect could also capture biological differences among the races that may make them more susceptible. Sixth, there are hospital-level characteristics not observed in the models that are absorbed by the type of hospital variable, which could bias the “barrier” effect itself.

## Conclusion

This article pointed to the need for investment in actions that guarantee equity in the Brazilian health system. To meet this aim, it is fundamental the adequate financing of the SUS and the strengthening of the participation of Black and Indigenous movements in the instances of social control of the system, such as in local, municipal, and state councils, and in the National Health Council, is fundamental to guarantee that their needs are considered in the multi-year Health Policies and that, indeed, these needs, which are also rights, are met.

Racism was an important social determinant of the health-disease process during the period under analysis in the study. The inclusion of themes such as racism and the health of historically exposed populations (e.g., Black/Biracial and Indigenous Populations) in the curricula of biomedical courses, as well as in the continuing education activities of health care workers, is fundamental to the reduction of health inequities. It is through education that the process of denaturalizing the racism instilled in our subjectivities begins.

## Supplementary Information

Below is the link to the electronic supplementary material.

Supplementary file1 (PDF 20 KB)

## Data Availability

Data are available in the public domain: SIVEP-GRIPE/MS (https://opendatasus.saude.gov.br/dataset/srag-2021-a-2023) and IBGE (https://censo2010.ibge.gov.br/).
