# Supplementary figures and images for: Racial Inequalities in the Health Establishment Access to the Treatment of COVID-19 in Brazil in 2020
Source: J Racial Ethn Health Disparities. 2024 Jan 8;12(1):222–33. doi: 10.1007/s40615-023-01866-1 (PMC11753332; doi:10.1007/s40615-023-01866-1)

## SIVEP/MoH

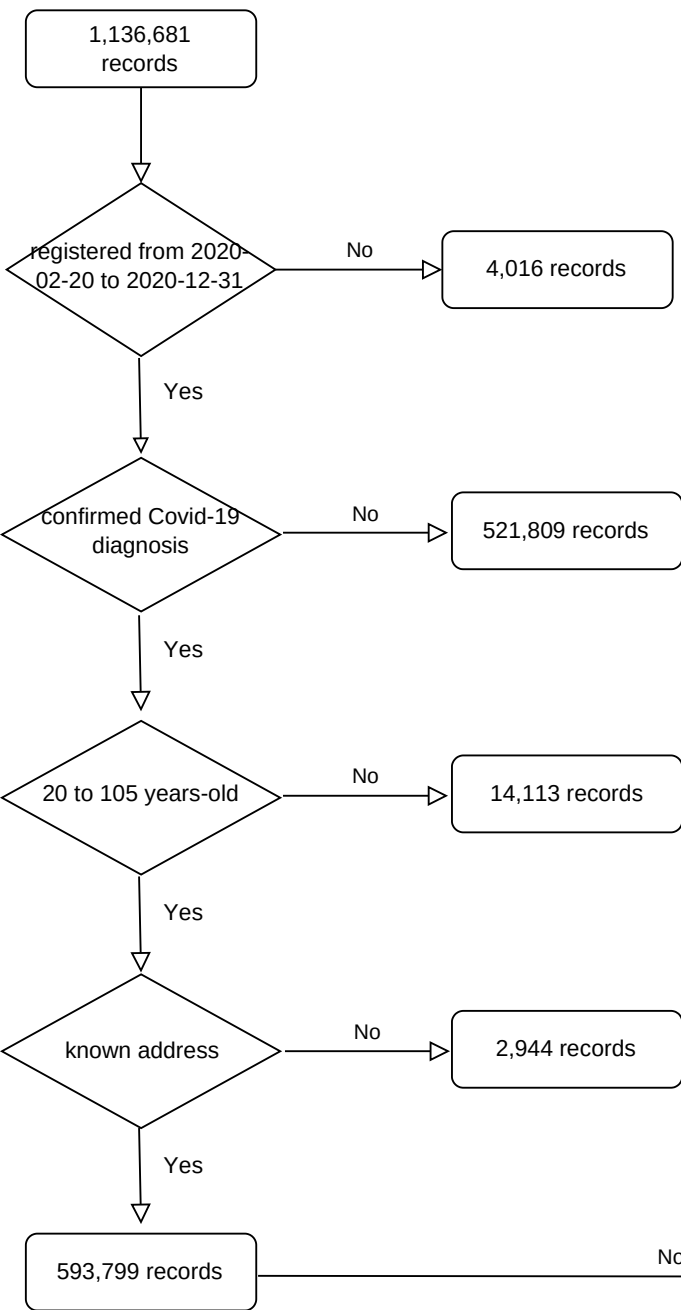

## CNES/MoH

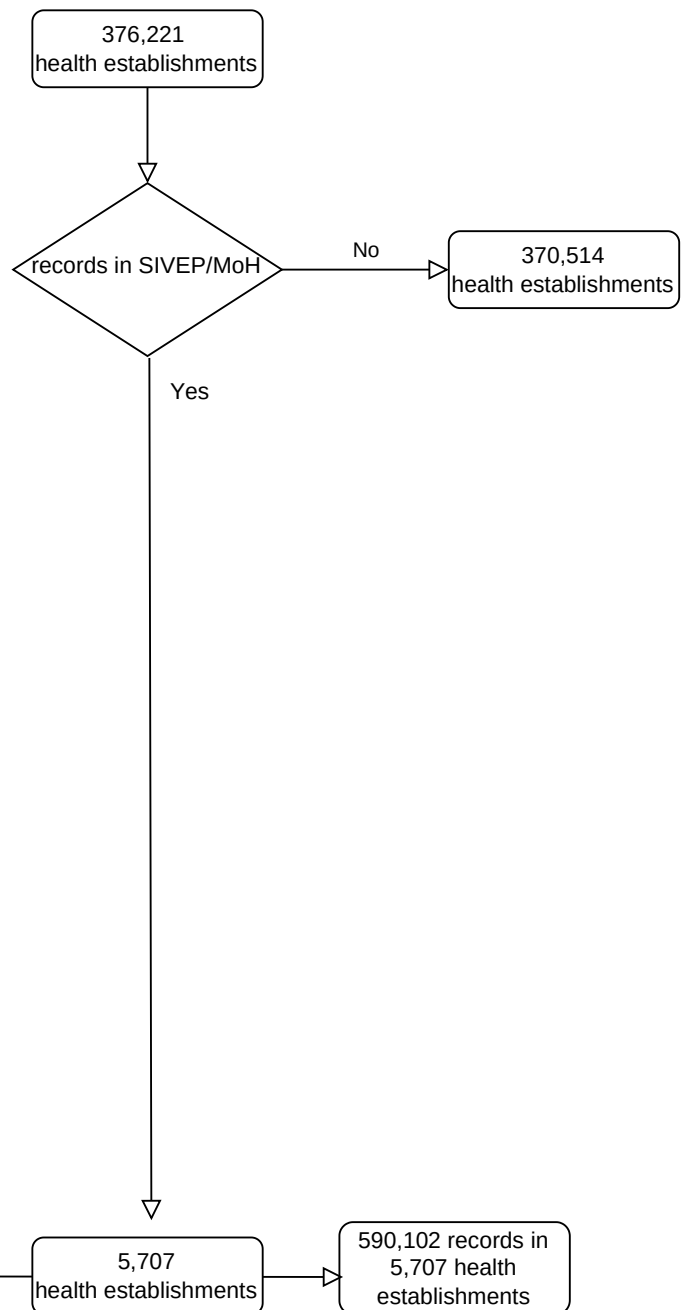

Supplement: Supplementary file 1 — Supplementary file1 (PDF 20 KB) [file 40615_2023_1866_MOESM1_ESM.pdf]
